# Comment: Why are females with Fabry disease affected?

**DOI:** 10.1016/j.ymgmr.2019.100529

**Published:** 2019-10-22

**Authors:** Michael Beck, Timothy M. Cox

**Affiliations:** a Institute of Human Genetics, University of Mainz, Mainz, Germany; b Department of Medicine, University of Cambridge, Cambridge, United Kingdom

**Keywords:** Fabry disease, Hunter syndrome, X-linked disorder, X-inactivation, Lysosomal storage disorder

Fabry disease (α-galactosidase deficiency, OMIM 301500) is an X-linked lysosomal disorder due to mutations in *GLA* and characterized by skin lesions (angiokeratomata), recurrent burning pain (acroparaesthesias), corneal clouding, hypohidrosis, cardiac and renal injury and cerebral ischaemia. Heterozygous females have variable clinical manifestations [1], and white-cell α-galactosidase activity may be deficient or within the healthy reference range. It has been assumed that severe disease in females who are heterozygous for an X-linked disorder is due to unfavourable skewing during Lyonization, so that there is biassed inactivation of the wild type allele in most tissues [2]. To determine whether preferential X-inactivation affecting the wild type *GLA* locus contributes to the pathological manifestations in Fabry heterozygotes, the validated clinical (Mainz) severity score values were compared with the X-inactivation status in 39 females. Heterozygotes in whom the inactivated X-chromosome carrying the wild-type parental *GLA* allele was dominant, were more severely affected than those without biassed X-inactivation [3]. These findings support the hypothesis that skewed X-inactivation is a major influence on the severity of clinical manifestations in Fabry heterozygotes. However, the question arises as to why, unlike female heterozygotes in Fabry disease, obligate heterozygous females in pedigrees affected by the X-linked Hunter syndrome (Mucopolysaccharidosis type II, OMIM 309900), only very exceptionally show clinical manifestations [4]. This lysosomal disease is also due to a deficiency of a soluble matrix enzyme of the lysosome, iduronate-2-sulphatase (IDS). Affected males have cardiovascular, respiratory, musculoskeletal manifestations that are often associated with progressive neurodegeneration [4]. De Camargo et al. analyzed clinical signs and symptoms, karyotype, pattern of X-inactivation, IDS activity, urinary glycosaminoglycan concentrations, computerized X-ray tomographic scans of abdomen and spine, and brain magnetic resonance imaging in 18 non-heterozygous and 22 heterozygous females. No difference between these groups, either on physical examination or spinal radiology, karyotype nor on the X-inactivation pattern was identified - although plasma and leukocyte IDS activities were significantly lower in plasma and leukocytes in the heterozygotes compared with healthy wild-type family members [5].

In the exceptionally rarely affected heterozygous females of Hunter syndrome, signs and symptoms can arise by distinct mechanisms such as structural abnormalities of the X-chromosome, homozygosity for mutant *IDS* alleles or markedly skewed X-inactivation that favours the X-chromosome bearing wild-type *IDS* allele [6]. All females with clinical manifestations of Hunter syndrome merit thorough investigation including karyotyping, analysis of X-inactivation pattern and determination of iduronate sulphatase activity. Pedigree analysis is mandatory, since as in Fabry disease, homozygosity can also be the cause of Hunter syndrome in a female [6].

Now the question arises why in Hunter syndrome (with a few exceptions) female heterozygotes are asymptomatic carriers and in Fabry disease heterozygous females often show clinical manfestations - albeit to a variable degree. One explanation is suggested by the results of cell culture experiments carried out by Fuller and co-workers [7]. Under the assumption that cross-correction of enzyme activity *in vivo* is ineffective in Fabry heterozygotes, the authors analyzed biosynthesis and secretion of α-galactosidase A in cultured fibroblasts, and determined enzyme activity in plasma. It was demonstrated that the proportion of α-galactosidase A that was secreted by unaffected fibroblasts into the culture media was significantly less than that of other lysosomal proteins. In control plasma, α-galactosidase A activity was similar to that of iduronate-2-sulphatase, however, the molecular form of α-galactosidase in plasma is the mature 46 kDa enzyme and not the high-uptake, mannose 6-phosphorylated form. Moreover, the authors confirmed that the mature 46 kDa enzyme, which lacks the mannose-6-phosphate residue cannot be efficiently endocytosed by affected cells. Using an artificial *in cellulo* technique, no complementary functional cross-correction (degradation of the storage material ceramide trihexoside) in the Fabry system was observed, whereas cross-correction (glycosaminoglycan degradation) was confirmed in Hunter fibroblasts.

These findings indicate that in contrast to the situation in Hunter syndrome, Fabry heterozygotes show clinical manifestation - and that *in vivo,* the unaffected cells principally secrete the mature, rather than the mannose-6-phosphorylated form of α-galactosidase that is able to complement the activity in the population of cells lacking expression of the enzyme. An alternative explanation is that compared with iduronate-2-sulphatase, α-galactosidase A released by the population of wild type cells in the mixed population of the female mosaic, is more susceptible to dephosphorylation by plasma phosphatases. This means that - by using the term that has been introduced by Dobyns et al. - in Fabry disease the gene product is operationally cell autonomous, as it cannot be readily complemented in the presence of wild type cells [8]. Under these circumstances, in females with α-galactosidase deficiency, the degree of disease expression with clinical manifestations will be more exquisitely dependent on the degree of X-inactivation - as unambiguously demonstrated by Echevarria et al. [9]: This group investigated 65 females with Fabry disease and explored the X-inactivation profiles in four tissues using DNA methylation analysis. The authors confirmed that heterozygous female Fabry patients with skewed X-inactivation profiles differed markedly in the severity of their clinical manifestations and in a manner that was directly related to which of the parenteral *GLA* alleles was most frequently inactivated: Inactivation of the mutant allele leads to a mild phenotype, and inactivation of the wild-type *GLA* allele induces disease with an earlier onset and worse prognosis [9].

In summary, it can be shown that in Fabry disease heterozygous females are not simply genetic carriers, but they express the pathological phenotype to a variable degree, as their plasma mostly contains 46 kDa mature form of the α-galactosidase A that lacking mannose 6-phosphorylated residues cannot readily be taken up by other cells. Enzyme replacement therapy, however, is at least partially effective as the recombinant enzyme preparations consist of the 52 kDa high-uptake form, containing numerous mannose 6-phosphorylated moieties.

In contrast to Fabry disease, in Hunter syndrome cross-correction of the metabolic defect takes place as - in comparison with α-galactosidase A - approximately 10-fold more iduronate-2-sulphatase is present in the culture medium. Furthermore, this enzyme is highly sialylated, a property which may maintain a circulating pool preventing receptor-mediated recapture and antibody recognition [7]. The findings provide a plausible operational explanation as to why heterozygous females with Hunter syndrome in general are simple genetic carriers without any clinical manifestations. Phenotypic expression in females with Hunter syndrome are exceptions that immediately mandate clarification by careful genetic studies (including karyotyping, degree of X-inactivation, pedigree analysis).

In summary, Fabry disease vividly shows that compared with Hunter disease, there is variable clinical expression in heterozygous females, as it is also seen in Danon disease (OMIM 300257), another X-linked disorders due to mutations in the lysosomal membrane protein LAMP-2B [10]. Thus as with many X-linked disorders, their pattern of inheritance cannot be categorised as an X-linked dominant or X-linked recessive trait. Moreover, it is clear that disease expression depends on many factors, including mutation, skewed X-inactivation, clonal expansion and somatic mosaicism. We concur with Dobyns et al., and also recommend that the terms X-linked recessive and dominant be discontinued, and that all such disorders be simply described as showing ‘X-linked inheritance‘ [8].
